# Autophagy Stimulation as a Potential Strategy Against Intestinal Fibrosis

**DOI:** 10.3390/cells8091078

**Published:** 2019-09-13

**Authors:** Jesus Cosin-Roger, Francisco Canet, Dulce C. Macias-Ceja, Laura Gisbert-Ferrándiz, Dolores Ortiz-Masiá, Juan V. Esplugues, Rafael Alós, Francisco Navarro, María D. Barrachina, Sara Calatayud

**Affiliations:** 1 Hospital Dr Peset, FISABIO, 46017 Valencia, Spain; 2 Centro de Investigación Biomédica en Red de Enfermedades Hepáticas y Digestivas, 46010 Valencia, Spain; 3 Departamento de Farmacología, Facultad de Medicina, Universidad de Valencia, 46010 Valencia, Spain; 4 Departamento de Medicina, Facultad de Medicina, Universidad de Valencia, 46010 Valencia, Spain; 5 Departamento de Cirugía del Aparato Digestivo, Hospital La Fe, 46526 Valencia, Spain; 6 Departamento de Cirugía y Coloproctología, Hospital de Manises, 46940 Valencia, Spain

**Keywords:** intestinal fibrosis, autophagy, inflammation

## Abstract

We recently observed reduced autophagy in Crohn’s disease patients and an anti-inflammatory effect of autophagy stimulation in murine colitis, but both anti- and pro-fibrotic effects are associated with autophagy stimulation in different tissues, and fibrosis is a frequent complication of Crohn’s disease. Thus, we analyzed the effects of pharmacological modulation of autophagy in a murine model of intestinal fibrosis and detected that autophagy inhibition aggravates, while autophagy stimulation prevents, fibrosis. These effects are associated with changes in inflammation and in collagen degradation in primary fibroblasts. Thus, pharmacological stimulation of autophagy may be useful against intestinal fibrosis.

## 1. Introduction

Crohn’s Disease (CD) is a chronic inflammatory pathology of the gut that in a significant proportion of patients, leads to complications related to the development of intestinal fibrosis and strictures that often need a surgical intervention.

Genetic studies identified several single nucleotide polymorphisms (SNPs) associated with CD in genes related to autophagy, a cellular process essential in intestinal homeostasis [1]. We reported that autophagy is reduced in the damaged mucosa of CD patients [2] and that autophagy stimulation prevents intestinal inflammation [3]. However, control of inflammation by the current therapies does not seem enough to prevent fibrosis and both anti- and pro-fibrotic effects have been attributed to autophagy stimulation in different organic systems [4,5]. Hence, we aim to analyze the effects of pharmacological modulation of autophagy in the development of intestinal fibrosis. 

## 2. Methods

### 2.1. Heterotopic Transplant Model of Intestinal Fibrosis

The murine intestinal fibrosis was induced in vivo by the heterotopic transplant of colonic tissue [6]. Briefly, colon resections of 1cm from C57BL/6 mice were washed with 0.9% NaCl and transplanted subcutaneously into the neck of recipient mice. After anesthetizing the mice with isoflurane, two perpendicular incisions to the body axis were made, the intestinal grafts were implanted and the incisions were closed with vicryl 5-stiches. We kept an adjacent segment of the colon from each donor mice as autologous control tissue. Recipient mice were treated with a daily intraperitoneal injection of Rapamycin (1.25 mg/kg), 3-methyladenine (3MA) (10 mg/kg) or its vehicle (DMSO 1%). Seven days after surgery, recipient mice were sacrificed by neck dislocation and intestinal grafts were collected. All tissues were subdivided for RNA and protein isolation and histological analysis. All protocols were approved by the institutional animal care and use committe of the University of Valencia, and all experiments were performed in compliance with the European Animal Research Laws (European Communities Council Directives 2010/63/EU, 90/219/EEC, Regulation (EC) No. 1946/2003) and Generalitat Valenciana (Artículo 31, Real Decreto 53/2013) (Ethical approval number 2018/VSC/PEA/0179, 28 August 2018).

### 2.2. Primary Intestinal Fibroblasts Isolation and Culture

Primary intestinal fibroblasts were obtained from the healthy tissue of intestinal resections from colon carcinoma patients [6]. Briefly, intestinal tissue was cut into 3-5mm pieces and epithelial cells were removed with an incubation with HBSS-EDTA of 30 min at 37 °C. Subsequently, small intestinal pieces were digested with collagenase I (1 mg/ml), DNAse (1 µl/ml) and hyaluronidase (2 mg/ml) during 30 min at 37 ºC. Finally, explants were maintained under culture with DMEM high glucose (Sigma-Aldrich) supplemented with FCS 20%, penicilin/streptomicin (100 µg/ml), gentamycin (100 µg/ml), amphotericin B (2 µg/ml), and ciprofloxacin (16 µg/ml) in a Petri dish. Primary intestinal fibroblasts were treated with TGF-β (5 ng/ml) and Rapamycin (50 nM) or Bafilomycin B1 (10 nM) during 24 h. The study was approved by the Institutional Review Board of both Hospital of Manises (Valencia, Spain) and Hospital La Fe (Valencia, Spain). Written informed consent was obtained from all participating patients.

### 2.3. RNA Extraction and Quantitative PCR

RNA from intestinal grafts/tissues and primary fibroblasts was isolated using the Illustra RNA Spin Mini (GE Healthcare). The tissue homogenization was performed with the gentleMACS™ Dissociator (Miltenyi Biotec), while primary fibroblasts were lysed with a 19G needle. cDNA was obtained after RT-PCR performed with the Prime Script RT reagent Kit (Takara Biotechnology). The expression of several genes (Table S1) was analyzed by quantitative PCR performed with the Prime Script Reagent Kit Perfect Real Time (Takara Biotechnology). Relative gene expression of each gene was expressed as follows: fold induction = 2 − Δ(ΔCT), where ΔCT = CT (target) − CT (housekeeping), and Δ(ΔCT) = ΔCT (treated) − ΔCT (control). In all cases β-actin was used as the housekeeping gene.

### 2.4. Western Blot 

Equal amounts of protein from intestinal grafts or primary intestinal fibroblasts were loaded onto SDS-PAGE gels. After electrophoresis and transference, membranes were blocked with 5% non-fat dry milk in TBS-T during 1 h at room temperature and incubated overnight at 4 °C with the primary antibody (Table S2). Afterwards, membranes were washed with TBS-T and incubated with a secondary antibody anti-mouse IgG (Thermo Scientific, 1:2500) or anti-rabbit IgG (Thermo Scientific, 1:5000) during 1 h at room temperature. Protein bands were detected by LAS-300 (Fujifilm) after treatment with SuperSignal West Pico Chemiluminescent substrate (Thermo Scientific). The densitometry of the bands was quantified with the software Image Gauge Version 4.0 (Fujifilm).

### 2.5. Sirius Red Staining

Sirius Red Staining was performed in 5 µm sections of paraffin-embedded colonic tissues in order to analyze the collagen layer in intestinal grafts obtained after the heterotopic transplant model. After deparaffinization and rehydration, slides were incubated with Fast green (Sigma-Aldrich) during 15 min at room temperature and with Sirius red 0.1% (Sigma-Aldrich) / Fast green 0.04% during 30 min at room temperature. Finally, tissues were dehydrated and visualized with a light microscope (1X81 Olympus).

### 2.6. Statistical Analysis

All data were expressed as mean ± S.E.M. and were compared by analysis of variance (one-way ANOVA) with a Newman–Keuls post hoc correction for multiple comparisons or a t-test when appropriate (Graph-Pad Software 6.0). A *p*-value <0.05 was considered to be statistically significant.

## 3. Results

**Figure 1:**
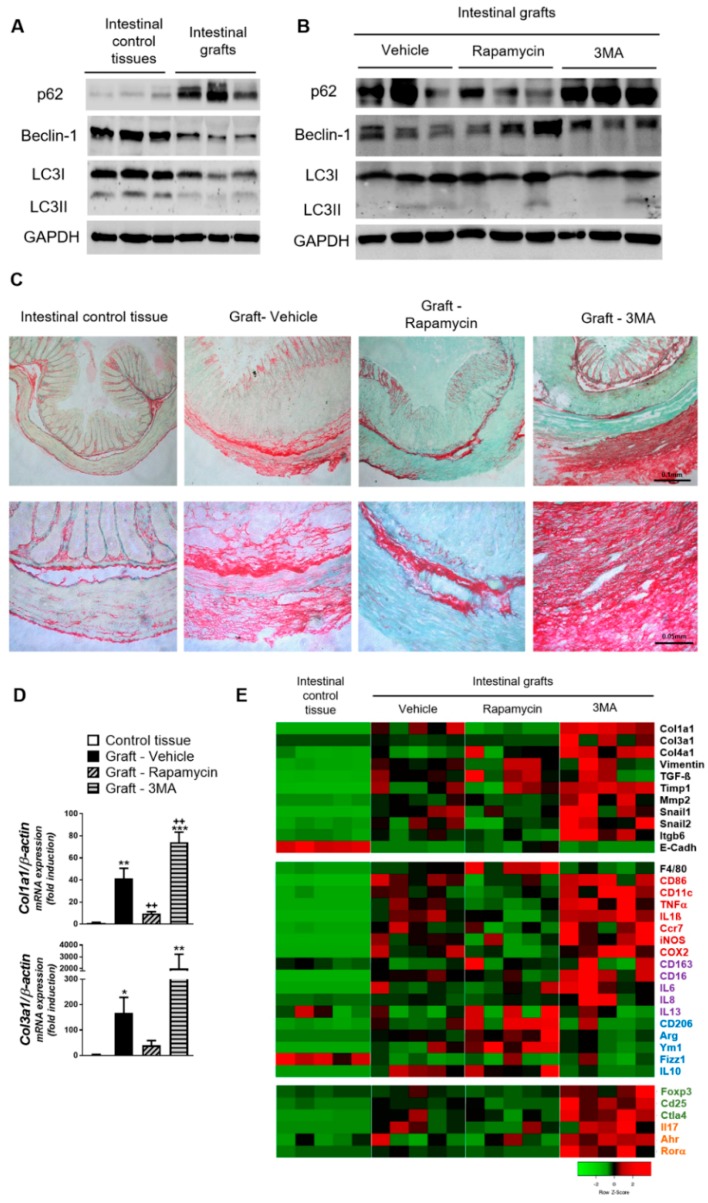
Murine fibrosis is reduced by autophagy stimulation and increased by autophagy inhibition. Murine intestinal fibrosis was induced by the heterotopic transplant of colonic tissue. Autophagy and fibrotic markers were analyzed in intestinal control tissues and the intestinal grafts resected seven days after transplantation from mice receiving a daily intraperitoneal injection of the autophagy stimulator rapamycin (1.25 mg/kg mice), the autophagy inhibitor 3-Methyladenine (3-MA, 10 mg/kg mice) or their vehicle (DMSO 1%, *n* = 5 each). (**A**) Representative Western blots showing the protein levels of the autophagy substrate P62, the autophagy protein Beclin-1, the two forms of LC3 (LC3-I, cytoplasmic; LC3-II, autophagosome-associated) and of Glyceraldehyde-3-Phosphate Dehydrogenase (GAPDH). The accumulation of P62 and the reduction in Beclin-1 and LC3-II in the explants from vehicle-treated mice indicate an inhibited autophagy that was partially prevented by rapamycin- and promoted by 3-MA- treatments (**B**). (**C**) Representative pictures of Sirius Red staining in paraffin-embedded tissues where the red color indicates collagen deposition (20× and 40× magnification in upper and lower panels respectively), and (**D**) mRNA expression of fibrous collagens (*Col1a1, Col3a1*) analyzed by qPCR (results normalized with β-actin and represented as fold induction vs. intestinal control tissues). (**E**) Heatmap showing the relative mRNA expression of genes involved in (i) fibrosis and epithelial to mesenchymal transition; (ii) inflammation: the general macrophage marker (F4/80), pro-inflammatory (red), anti-inflammatory (blue) and pro-fibrotic (purple) molecules; and (iii) T lymphocyte markers (regulatory T cells—green, Th17—orange). Bars in graphs represent mean ± S.E.M. Significant differences vs. control intestinal tissues are shown by * *p* < 0.05, ** *p* < 0.01 and *** *p* < 0.001; significant differences vs. grafts obtained from vehicle-treated mice are shown by ++ *p* <0.01; as analyzed by ANOVA with a Newman–Keuls post hoc correction for multiple comparisons (Graph-Pad Software v6.0).

Seven days after implantation, grafts exhibited a significant inhibition of autophagy as shown by the accumulation of p62 and the reduction of LC3II and Beclin-1 (Figure 1A). As expected, rapamycin stimulated and 3-MA further inhibited autophagy in the grafted tissue (Figure 1B).

Explanted colon tissues presented a significant deposition of collagen in the mucosa, submucosa and subserosa. Of interest, treatment of mice with rapamycin significantly reduced the collagen layer thickness and the contrary occurred in tissues resected from 3-MA-treated mice (Figure 1C). These results were strongly reinforced by the gene expression of pro-fibrotic markers. The fibrotic grafts from vehicle-treated mice showed a significantly increased expression of the two fibrous collagens (Col1a1, Col3a1) (Figure 1D), and of other molecules associated with fibrosis and epithelial to mesenchymal transition (Vimentin, TGFß, Timp1, Mmp2, Snail1, Snail2, Itgb6) with a parallel reduction in the epithelial marker E-cadherin. Grafts from mice receiving rapamycin showed reduced mRNA expression of these collagens and a partial reversion on the E-cadherin reduction, while the other parameters were not significantly modified. Finally, tissues from 3-MA-treated animals showed a significant up-regulation of most pro-fibrotic genes with regard to the expression observed in vehicle-treated mice (Figure 1D,E).

**Figure 2:**
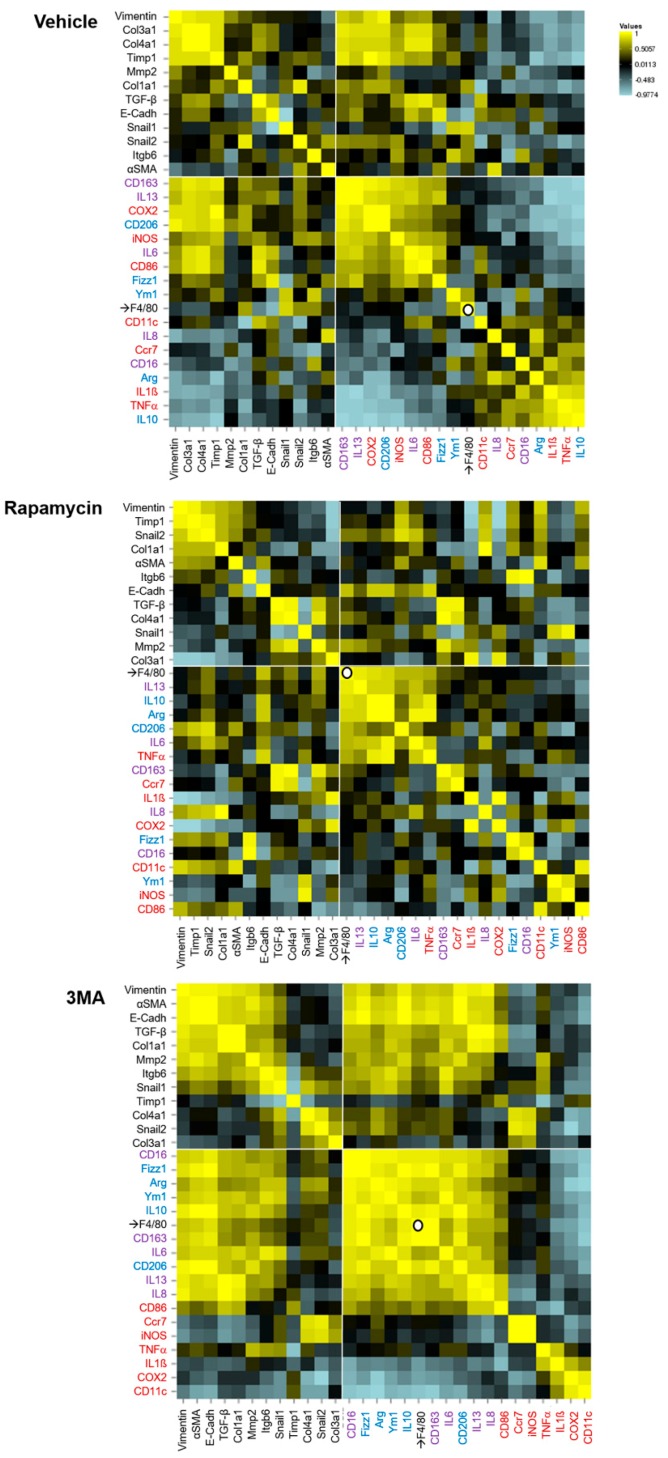
Correlation matrixes representing the Pearson’s correlation coefficient between pairs of data on mRNA expression in intestinal explants. Murine intestinal fibrosis was induced by the heterotopic transplant of colonic tissue in mice receiving a daily intraperitoneal injection of the autophagy stimulator rapamycin (1.25 mg/kg mice), the autophagy inhibitor 3-methyladenine (3-MA, 10 mg/kg mice) or their vehicle (DMSO 1%, *n* = 5 each). The intestinal grafts resected seven days after transplantation were analyzed with regard to their mRNA expression of genes involved in (i) fibrosis and epithelial to mesenchymal transition; and (ii) inflammation: the general macrophage marker (F4/80), pro-inflammatory (red), anti-inflammatory (blue) and pro-fibrotic (purple) molecules. The data were organized taking as references: (i) the fibroblast marker vimentin (in all groups); and (ii) the macrophage marker which correlates with a higher number of markers of inflammation in each experimental group (vehicle: CD163; rapamycin: F4/80, 3-MA: CD16). The pairwise comparison heatmaps were performed using the online resource available at http://www.heatmapper.ca, and interpreted according to its authors’ instructions [7].

In line with our previous study [6], grafts obtained from vehicle-treated mice showed an increased expression of macrophage markers, cytokines and other modulators of inflammation. We observed an up-regulation of pro-inflammatory (red), anti-inflammatory (blue) and pro-fibrotic (purple) molecules. These tissues also presented an increased expression of T lymphocyte markers (regulatory T cells—green, Th17—orange). Rapamycin treatment increased macrophage infiltration (F4/80 up-regulation) and the expression of anti-inflammatory agents. On the contrary, 3-MA promoted the expression of pro-inflammatory and pro-fibrotic mediators as well as that of T cell markers (Figure 1E). From the analysis of these results in a correlation matrix we deduce that in 3-MA treated mice, CD16-expression is associated with that of several markers of regulatory/pro-fibrotic macrophages that in turn, correlate with the expression of most fibrotic indicators. The definition of a predominant macrophage phenotype associated with fibrosis is less clear in animals treated with vehicle, and completely absent in rapamycin-treated animals (Figure 2). 

**Figure 3:**
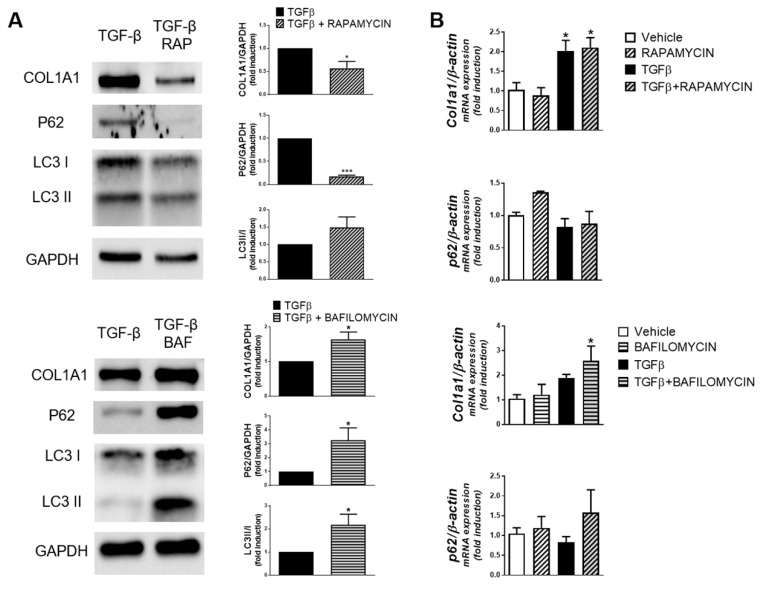
Human primary fibroblasts isolated from the healthy margin of intestinal carcinoma resections (*n* = 5) were treated with TGF-β (5 ng/ml), in the presence of Rapamycin (50 nM), Bafilomycin B1 (10 nM) or their vehicles, for 24 h. (**A**) Representative Western blots and protein levels of Col1a1, P62, LC3-I/II and GAPDH; and (**B**) graphs showing *Col1a1* and *P62* mRNA expression (results normalized with β-actin and represented as fold induction vs. vehicle-treated cells). Bars in graphs represent mean ± S.E.M. Significant differences vs. vehicle-treated fibroblasts are shown by * *p* < 0.05, and *** *p* < 0.001; as analyzed by ANOVA with a Newman–Keuls post hoc correction for multiple comparisons or a *t*-test when appropriate (Graph-Pad Software v6.0).

Of interest, in human primary fibroblasts treated with TGF-β, the increase in autophagy induced by rapamycin was associated with a reduction in the Col1a1 protein while autophagy blockade with bafilomycin-B1 provoked its accumulation (Figure 3A). None of these treatments modified Col1a1 mRNA expression (Figure 3B).

## 4. Discussion

This study demonstrates a reduced autophagy in murine intestinal fibrosis. Autophagy stimulation exerts an anti-fibrotic effect and enhances collagen degradation in fibroblasts, while autophagy inhibition aggravates fibrosis in association with significant changes in the inflammatory response and a reduction in collagen digestion.

Fibrosis has been linked with both increased and defective autophagy in different organic contexts [4,5]. In the intestine, SNPs in genes of autophagy predispose to inflammatory bowel disease (IBD) and their presence is associated with the development of stricturing and penetrating complications [8,9]. In coherence with this clinical data, the development of fibrosis in our murine model is associated with an inhibition of autophagy and the contribution of a defective autophagy to fibrosis is further substantiated by the worsening effect induced by the autophagy inhibitor 3-MA. Of significance, the stimulation of autophagy with rapamycin prevents this pathological process.

We observed that the inflammatory response that accompanied fibrosis was significantly affected by autophagy modulation. In vehicle-treated animals, the increased macrophage infiltrate seems to include both pro-inflammatory and anti-inflammatory/regulatory phenotypes. In contrast, in 3-MA-treated mice, we encountered an increased expression of pro-inflammatory mediators and a well-defined macrophage phenotype characterized by CD16-expression that, as seen before [10], seems relevant for the fibrotic process. These macrophages, together with CD16, express other classic M2 markers and are probably the source of the pro-fibrotic cytokines IL6, IL13, and IL8 [11,12]. The increase in fibrosis may also be related to the higher infiltration of Th17 and regulatory T cells observed in these tissues [13,14]. Finally, stimulation of autophagy with rapamycin increased the expression of anti-inflammatory mediators, as occurred in colitis [3], and augmented the influx of macrophages that seem of a regulatory/anti-inflammatory profile. In line with this, an anti-fibrotic effect of autophagy stimulation and the consequent inhibition of the IL-22/IL-23 axis in macrophages has been recently demonstrated in a different model of intestinal fibrosis [15]. Finally, we observed that treatment of isolated primary fibroblasts with rapamycin or the autophagy blocker bafilomycin significantly decreased or increased, respectively, collagen protein levels without affecting its gene expression. This implies that fibroblast’s autophagy, and its pharmacological regulation, affects collagen degradation. This mechanism most probably contributes to the effects of rapamycin and 3-MA on fibrosis in vivo and would add to the regulation of collagen gene expression observed in the colonic grafts. Thus, autophagy stimulation seems to inhibit intestinal fibrosis by modulating the function of the innate immune system and the mesenchymal activity.

In summary, autophagy inhibition seems relevant in the development of intestinal fibrosis and the pharmacological activation of autophagy constitutes a promising strategy against this CD complication.

## Supplementary Materials

The following are available online at https://www.mdpi.com/2073-4409/8/9/1078/s1, Table S1. Primer sequences of specific PCR products for each gene analysed. Table S2. Primary antibodies used in Western Blot analysis.

## References

[1] Kim S., Eun H.S., Jo E.K. (2019). Roles of Autophagy-Related Genes in the Pathogenesis of Inflammatory Bowel Disease. Cells.

[2] Ortiz-Masia D., Cosin-Roger J., Calatayud S., Hernandez C., Alos R., Hinojosa J., Apostolova N., Alvarez A., Barrachina M.D. (2014). Hypoxic macrophages impair autophagy in epithelial cells through Wnt1: Relevance in IBD. Muc. Immunol..

[3] Macias-Ceja D.C., Cosin-Roger J., Ortiz-Masia D., Salvador P., Hernandez C., Esplugues J.V., Calatayud S., Barrachina M.D. (2017). Stimulation of autophagy prevents intestinal mucosal inflammation and ameliorates murine colitis. Br. J. Pharmacol..

[4] Del Principe D., Lista P., Malorni W., Giammarioli A.M. (2013). Fibroblast autophagy in fibrotic disorders. J. Pathol..

[5] Hilscher M., Hernandez-Gea V., Friedman S.L. (2012). Autophagy and mesenchymal cell fibrogenesis. Biochim. Biophys. Acta.

[6] Macias-Ceja D.C., Ortiz-Masia D., Salvador P., Gisbert-Ferrandiz L., Hernandez C., Hausmann M., Rogler G., Esplugues J.V., Hinojosa J., Alos R. (2019). Succinate receptor mediates intestinal inflammation and fibrosis. Mucosal. Immunol..

[7] Babicki S., Arndt D., Marcu A., Liang Y., Grant J.R., Maciejewski A., Wishart D.S. (2016). Heatmapper: Web-enabled heat mapping for all. Nucleic Acids Res..

[8] Fowler E.V., Doecke J., Simms L.A., Zhao Z.Z., Webb P.M., Hayward N.K., Whiteman D.C., Florin T.H., Montgomery G.W., Cavanaugh J.A. (2008). ATG16L1 T300A shows strong associations with disease subgroups in a large Australian IBD population: further support for significant disease heterogeneity. Am. J. Gastroenterol..

[9] Strisciuglio C., Auricchio R., Martinelli M., Staiano A., Giugliano F.P., Andreozzi M., De Rosa M., Giannetti E., Gianfrani C., Izzo P. (2014). Autophagy genes variants and paediatric Crohn’s disease phenotype: A single-centre experience. Dig. Liver. Dis..

[10] Salvador P., Macias-Ceja D.C., Gisbert-Ferrandiz L., Hernandez C., Bernardo D., Alos R., Navarro-Vicente F., Esplugues J.V., Ortiz-Masia D., Barrachina M.D. (2018). CD16+ Macrophages Mediate Fibrosis in Inflammatory Bowel Disease. J. Crohns Colitis.

[11] Chang J., Hisamatsu T., Shimamura K., Yoneno K., Adachi M., Naruse H., Igarashi T., Higuchi H., Matsuoka K., Kitazume M.T. (2013). Activated hepatic stellate cells mediate the differentiation of macrophages. Hepatol. Res..

[12] Liaskou E., Zimmermann H.W., Li K.K., Oo Y.H., Suresh S., Stamataki Z., Qureshi O., Lalor P.F., Shaw J., Syn W.K. (2013). Monocyte subsets in human liver disease show distinct phenotypic and functional characteristics. Hepatology.

[13] Ray S., De Salvo C., Pizarro T.T. (2014). Central role of IL-17/Th17 immune responses and the gut microbiota in the pathogenesis of intestinal fibrosis. Curr. Opin. Gastroenterol..

[14] Filidou E., Valatas V., Drygiannakis I., Arvanitidis K., Vradelis S., Kouklakis G., Kolios G., Bamias G. (2018). Cytokine Receptor Profiling in Human Colonic Subepithelial Myofibroblasts: A Differential Effect of Th Polarization-Associated Cytokines in Intestinal Fibrosis. Inflamm. Bowel. Dis..

[15] Mathur R., Alam M.M., Zhao X.F., Liao Y., Shen J., Morgan S., Huang T., Lee H., Lee E., Huang Y. (2019). Induction of autophagy in Cx3cr1(+) mononuclear cells limits IL-23/IL-22 axis-mediated intestinal fibrosis. Mucosal. Immunol..

