# Supplementary material for: Autophagy Stimulation as a Potential Strategy Against Intestinal Fibrosis
Source: Cells. 2019 Sep 13;8(9):1078. doi: 10.3390/cells8091078 (PMC6770118; doi:10.3390/cells8091078)
Supplement: Supplementary file 1 [file cells-08-01078-s001.pdf]

SUPPLEMENTARY INFORMATION

Table S1. Primer sequences of specific PCR products for each gene analyzed

Gene	Sense (5´-3´)	Antisense (5´-3´)	Length (bp)
**Mouse**			
Col1a1	CAGGCTGGTGTGATGGGATT	AAACCTCTCTCGCCTCTTGC	317
Col3a1	AGTGGGCATCCAGGTCCTAT	GTGCTTACGTGGGACAGTCA	480
Col4a1	TCCTCACTGTGGATCGGCTA	TTGGATCAGGAGCGCCATTT	554
Vimentin	GCTCCTACGATTCACAGCCA	CGTGTGGACGTGGTCACATA	190
TGF-β	GCGGACTACTATGCTAAAGAGG	TCAAAAGACAGCCACTCAGG	295
Timp1	CTTGGTTCCCTGGCGTACTC	ACCTGATCCGTCCACAAACAG	149
Mmp2	CAAGTTCCCCGGCGATGTC	TTCTGGTCAAGGTCACCTGTC	170
Snail1	ATGCACATCCGAAGCCACAC	GGTCAGCAAAAGCACGGTTG	148
Snail2	GAAGCCCAACTACAGCGAAC	ATAGGGCTGTATGCTCCCGA	123
Itgb6	ATGTGCTTTTTCAGGGATGGTG	GCACGGGAGACCCTCTAATC	143
E-Cadherin	AACCCAAGCACGTATCAGGG	ACTGCTGGTCAGGATCGTTG	142
F4/80	TGACTCACCTTGTGGTCCTAA	CTTCCCAGAATCCAGTCTTTCC	110
CD86	GCACGGACTTGAACAACCAG	CCTTTGTAAATGGGCACGGC	194
CD11c	TCTTCTGCTGTTGGGGTTTG	CAGTTGCCTGTGTGATAGCC	204
TNF-α	GATCGGTCCCCAAAGGGATG	GGTGGTTTGTGAGTGTGAGGG	86
IL-1β	TGCCACCTTTTGACAGTGATG	ATGTGCTGCTGCGAGATTTG	136
Ccr7	CTCTCCACCGCCTTTCCTG	ACCTTTCCCCTACCTTTTTATTCCC	126
iNOS	CGCTTGGGTCTTGTTCACTC	GGTCATCTTGTATTGTTGGGCTG	222
COX-2	CCCGGACTGGATTCTATGGTG	TTCGCAGGAAGGGGATGTTG	153
CD163	GACACACGGAGCCATCAAAATC	TCACAGCCACAACAAAGAAACCT	126
CD16	GAAGGGGAAACCATCACGCT	GCAAACAGGAGGCACATCAC	293
IL-6	GAGTCCTTCAGAGAGATACAGAAAC	TGGTCTTGGTCCTTAGCCAC	150
IL-8	CTGCTGGCTGTCCTTAACC	TCTGTTGCAGTAAATGGTCTCG	150
IL-13	GCCAAGATCTGTGTCTCTCCC	ACTCCATACCATGCTGCCG	106
CD206	TGTGGAGCAGATGGAAGGTC	TGTCGTAGTCAGTGGTGGTTC	201
Arginase	GTGGGGAAAGCCAATGAAGAG	TCAGGAGAAAGGACACAGGTTG	232
Ym1	AGAAGCAATCCTGAAGACACC	GCATTCCAGCAAAGGCATAG	205
Fizz1	CGTGGAGAATAAGGTCAAGGAAC	CAACGAGTAAGCACAGGCAG	212
IL-10	GGACAACATACTGCTAACCGAC	CCTGGGGCATCACTTCTACC	112
Foxp3	GTATTGAGGGTGGGTGTCAGG	ACAGCATGGGTCTGTCTTCTC	95
Cd25	TGAAGTGTGGGAAAACGGGG	TTGTGGGAAGTCTGTGGTGG	158
Ctla4	GGACTTGGCCTTTTGTAGCC	ACACCACTGAAGGTTGGGTC	87
Il17	GCAAACATGAGTCCAGGGAGAG	CAGGGTCTTCATTGCGGTGG	262
Ahr	TGTTGCATTAAAGTCCACCCC	ACCAGCACAAAGCCATTCAG	129
Rorα	GCGGCGTAAAGGATGTATTTTG	TGCTCTGCTGACTTCTCCTG	154
β-actin	GCCAACCGTGAAAAGATGACC	GAGGCATACAGGGACAGCAC	95
**Human**			
Col1a1	GGAGCAGACGGGAGTTTCTC	CCGTTCTGTACGCAGGTGAT	252
P62	CCGTGAAGGCCTACCTTCTG	TCCTCGTCACTGGAAAAGGC	214
β-actin	GGACTTCGAGCAAGAGATGG	AGCACTGTGTTGGCGTACAG	57

Table S2. Primary antibodies used in Western Blot analysis

Antibody	Dilution
Anti-SQSTM1 (Santa Cruz, SC-28359)	1:1000
Anti-Beclin 1 (Novus Biologicals, NB500-249)	1:1000
Anti-Col1a1 (Cell Signalling, 84336S)	1:1000
Anti-LC3 (Sigma-Aldrich, L8918)	1:1000
Anti-GAPDH (Sigma-Aldrich, G9545)	1:5000
